# Detection of Newcastle disease virus and assessment of associated relative risk in backyard and commercial poultry in Kerala, India

**DOI:** 10.1002/vms3.747

**Published:** 2022-02-24

**Authors:** Chintu Ravishankar, Rajasekhar Ravindran, Anneth Alice John, Nithin Divakar, George Chandy, Vinay Joshi, Deepika Chaudhary, Nitish Bansal, Renu Singh, Niranjana Sahoo, Sunil K. Mor, Nand K. Mahajan, Sushila Maan, Naresh Jindal, Megan A. Schilling, Catherine M. Herzog, Saurabh Basu, Jessica Radzio‐Basu, Vivek Kapur, Sagar M. Goyal

**Affiliations:** ^1^ Department of Veterinary Microbiology, College of Veterinary and Animal Sciences, and Centre for Wildlife Studies Kerala Veterinary and Animal Sciences University Pookode Kerala India; ^2^ Departments of Veterinary Public Health and Epidemiology and Animal Biotechnology, College of Veterinary Sciences Lala Lajpat Rai University of Veterinary and Animal Science Hisar India; ^3^ College of Veterinary Science and Animal Husbandry Orissa University of Agriculture and Technology Bhubaneswar Odisha India; ^4^ Veterinary Population Medicine Department University of Minnesota St. Paul Minnesota; ^5^ Department of Animal Sciences The Pennsylvania State University University Park Pennsylvania; ^6^ The Huck Institutes of the Life Sciences The Pennsylvania State University University Park Pennsylvania; ^7^ Department of Industrial and Manufacturing Engineering College of Engineering The Pennsylvania State University University Park Pennsylvania

**Keywords:** F gene, M gene, Newcastle disease, poultry, prevalence, relative risk, RT‐PCR

## Abstract

**Background:**

Newcastle disease (ND) is an economically important viral disease affecting the poultry industry. In Kerala, a state in South India, incidences of ND in commercial and backyard poultry have been reported. But a systematic statewide study on the prevalence of the disease has not been carried out.

**Objectives:**

A cross‐sectional survey was performed to detect the presence of Newcastle disease virus (NDV) in suspect cases and among apparently healthy commercial flocks and backyard poultry, in the state and to identify risk factors for NDV infection.

**Methods:**

Real‐time reverse transcription‐PCR (RT‐PCR) was used to detect the M gene of NDV in choanal swabs and tissue samples collected from live and dead birds, respectively and the results were statistically analysed.

**Results:**

The predominant clinical signs of the examined birds included mild respiratory signs, huddling together and greenish diarrhoea. Nervous signs in the form of torticollis were noticed in birds in some of the affected flocks. On necropsy, many birds had haemorrhages in the proventriculus and caecal tonsils which were suggestive of ND. Of the 2079 samples tested, 167 (8.0%) were positive for the NDV M‐gene by RT‐PCR. Among 893 samples collected from diseased flocks, 129 (14.5%), were positive for M gene with pairwise relative risk (RR) of 15.6 as compared to apparently healthy flocks where 6 out of 650 (0.9%) samples were positive. All positive samples were from poultry; none of the ducks, pigeons, turkey and wild birds were positive. Commercial broilers were at higher risk of infection than commercial layers (RR: 4.5) and backyard poultry (RR: 4.9). Similarly, birds reared under intensive housing conditions were at a higher risk of being infected as compared to those reared under semi‐intensive (RR: 6.7) or backyard housing (RR: 2.1). Multivariable analysis indicated that significantly higher risk of infection exists during migratory season and during ND outbreaks occurring nearby. Further, lower risk was observed with flock vaccination and backyard or semi‐intensive housing when compared to intensive housing. When the M gene positive samples were tested by RT‐PCR to determine whether the detected NDV were mesogenic/velogenic, 7 (4.2%) were positive.

**Conclusions:**

In Kerala, NDV is endemic in poultry with birds reared commercially under intensive rearing systems being affected the most. The outcome of this study also provides a link between epidemiologic knowledge and the development of successful disease control measures. Statistical analysis suggests that wild bird migration season and presence of migratory birds influences the prevalence of the virus in the State. Further studies are needed to genotype and sub‐genotype the detected viruses and to generate baseline data on the prevalence of NDV strains, design better detection strategies, and determine patterns of NDV transmission across domestic poultry and wild bird populations in Kerala.

## Abstract 2

A study was carried out to detect Newcastle disease virus in commercial and backyard chicken in Kerala, India, by employing real time RT‐PCR. The overall percentage positivity obtained was 8%. Risk analysis revealed significantly higher risk for broiler birds and intensive type of housing. The risk was also higher for birds housed in facilities in areas with a history of occurrence of the disease, if migratory birds were present in the area, and during bird migration season. It was also observed that vaccination had a protective effect as indicated by lower relative risk values.

## Graphical abstract

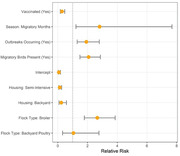

## 1 INTRODUCTION

Poultry production is an important contributor to agricultural productivity in India. Domestic poultry is reared for meat and eggs in large commercial farms as well as in small‐scale backyard units. As in any livestock industry, infectious diseases pose a major threat to poultry production. One such disease is Newcastle disease (ND), which is caused by virulent strains of *Avian orthoavulavirus*‐1 (commonly known as *Avian Paramyxovirus*‐1 (APMV‐1) or Newcastle disease virus (NDV)), a negative‐sense single‐stranded RNA virus of the genus *Orthoavulavirus*, family *Paramyxoviridae* (Dimitrov et al., 2019; ICTV, 2019). Based on pathogenicity to chicken embryos, strains of NDV are designated as lentogenic, mesogenic or velogenic, ordered by increasing virulence. In the United States, virulent NDV is regarded as a pathogen of national concern and a significant threat to animal agriculture (Brown & Bevins, 2017). On the basis of disease produced in chickens under laboratory condition, NDV isolates have been placed in five pathotypes; viscerotropic‐velogenic, neurotropic‐velogenic, mesogenic, lentogenic and asymptomatic enteric (Alexander, 2011).

NDV has been classified into Class I and Class II on the basis of its genome sequence (Czeglédi et al., 2006). Class I viruses circulate mostly in wild birds and are less virulent as compared to Class II viruses, which are more prevalent among domestic poultry. According to a recent classification scheme based on the complete fusion (F) gene sequence of the virus, Class I NDV is grouped under a single genotype (Genotype I) having three sub‐genotypes, while class II viruses are grouped into at least 20 distinct genotypes (I to XXI) with the exception of genotype XV that contains only recombined NDV viruses. Of these Class II genotypes, genotypes I, V, VI, VII, XII, XIII, XIV, XVIII and XXI are further divided into sub‐genotypes (Dimitrov et al., 2019).

The disease caused by velogenic NDV strains is included in the World Animal Health Organization [Office International des Epizooties (OIE)] list of avian diseases, is reportable, and is controlled in many countries by regular vaccination. NDV can infect at least 241 species of birds, representing 27 of the 50 orders of *Aves* (Aldous et al., 2010). Cormorants, pigeons and psittacine birds are commonly infected with NDV and are known to serve as a source of virulent virus to domestic poultry. Strains of low virulence (lentogenic strains) are also prevalent in poultry and wild birds including waterfowl (Miller, 2014; Brown & Bevins, 2017).

In India, the poultry sector is valued at about 800 billion rupees (2015–2016) (approximately 11 billion USD) and the highly organised commercial sector contributes to a majority (about 80%) of the total market share (DAHDF, 2017). ND was first reported in India in 1928 and is currently present in most of the country. The first outbreak of ND in India occurred in 1927 in Ranikhet, Uttarakhand in North India and there have been several incidences of the disease in the country since then. Several reports on the isolation and characterisation of NDV from different parts of the country have also been published (Ananth et al., 2008; Gowthaman et al., 2019; Kumanan et al., 1992; Morla et al., 2016; Roy et al. 2000; Tirumurugaan et al., 2011). Control efforts include regular vaccination of commercial poultry; however, backyard poultry are usually not vaccinated, which often leads to free circulation of the virus in these birds. The estimated total economic losses from January 2013 to July 2014 among commercial layers in Gujarat state of India was 3.7 million rupees (approximately 50,500 USD) (Khorajiya et al., 2018).

Being in the tropics, Kerala has a humid wet climate with seasonal monsoons. The state has both commercial and backyard poultry units. Common diseases present in Kerala poultry are ND, infectious bronchitis (Fathima et al., 2018; Ravishankar et al., 2015), infectious bursal disease (Nandhakumar et al., 2020), fowl cholera and salmonellosis (Ravishankar et al., 2008). Of these, ND is economically important because outbreaks of this disease occur more frequently. In Kerala, the virus has been isolated from Indian mynah (*Acridotheres tristis*) (Sulochana et al., 1982), pigeons (Sulochana & Mathew, 1991) and Japanese quails (Mini et al., 2001) and chicken (Arun, 2004). The virus has also been detected in pigeons along with other viral and bacterial pathogens (Dhivahar et al., 2018; Reji et al., 2017). However, no systematic statewide study has been carried out to map the prevalence of NDV. This cross‐sectional survey was performed to better estimate NDV prevalence in Kerala and identify risk factors for NDV infection among domestic poultry under varying production systems.

## 2 MATERIALS AND METHODS

### 2.1 Source of samples

**FIGURE 1:**
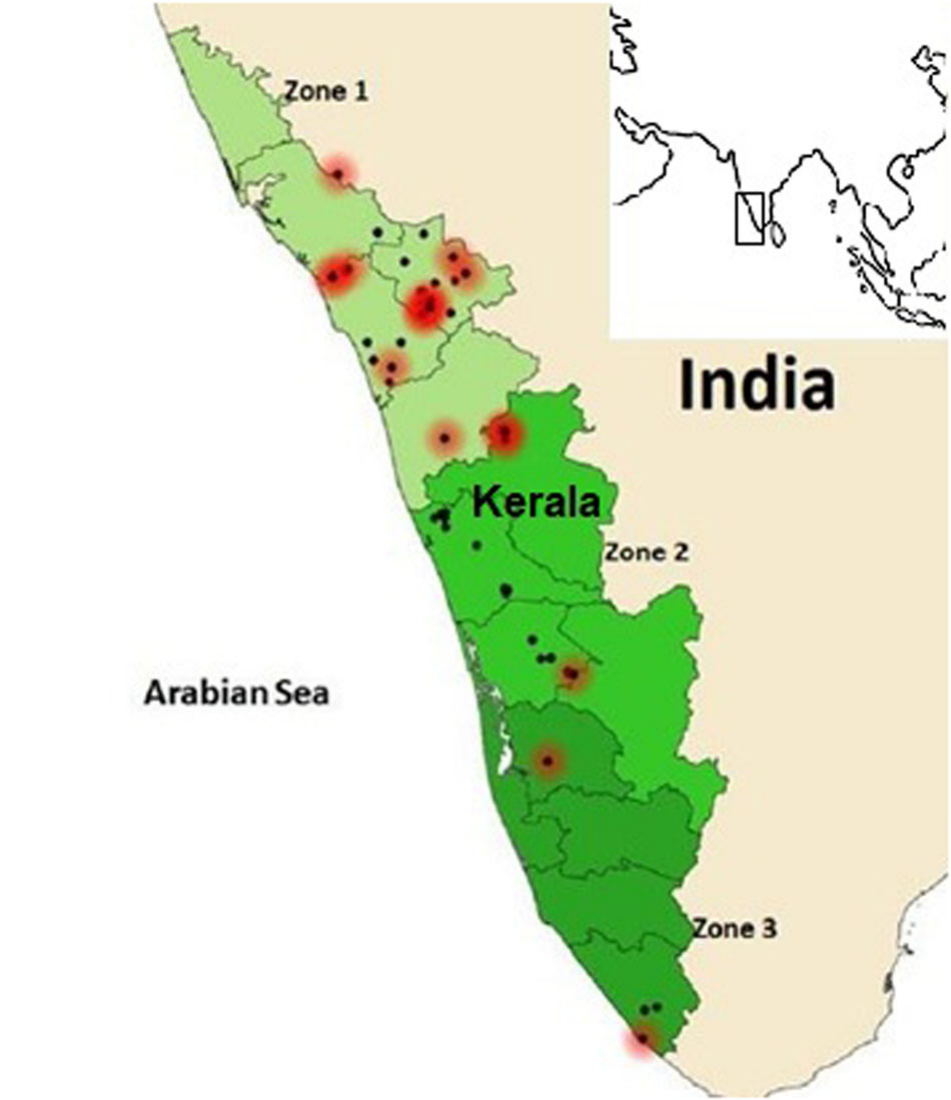
Different sampling zones in Kerala with locations of sampling sites and positive cases. Dark spot indicates sampling locations and red halo indicates positive locations

The state of Kerala was arbitrarily divided into three zones based on area (km^2^) and geographic location (Figure 1). A total of 70 facilities (farm or a household) housing 132 flocks were sampled between February 2018 and March 2019. Of the 70 facilities, a majority (n = 43) were layer facilities. Samples were also taken from broiler (*n* = 7), backyard (*n* = 3) and duck (*n* = 1) facilities and from facilities having mixed types of flocks (layer and backyard; *n* = 15). Migratory/wild birds were sampled from a single location only. Choanal swabs (*n* = 1930) from live birds and tissue samples (*n* = 149) such as lungs, trachea and spleen from dead birds suspected of NDV were collected from diverse avian species in each of the three zones across Kerala. The number of flocks per facility ranged from 1 through 9. The median and mean sampling density were 0.115 and 0.21, respectively (Supplementary Table 1). Samples were stratified based on the categories listed in Table 1. Clinical signs, vaccination history and age of the birds were recorded along with details of geographical location using EpiCollect5 (https://five.epicollect.net/). Vaccinated birds have been either administered the live lentogenic vaccine, LaSota, or the live mesogenic vaccine, R2B/Mukteswar. Few birds had been vaccinated with inactivated ND vaccine (Supplementary Table 2). Flock status at the time of sampling was recorded as healthy, having mild respiratory signs, or diseased (mortality ∼0.5%). Individual bird status was recorded as apparently healthy, sick (birds in which clinical signs such as respiratory distress, ruffled feathers were observed at time of sampling), recovered (according to owner self‐report) or dead (haemorrhages in the proventriculus and caecal tonsils were taken as suggestive of NDV infection). Recovered birds were from sheds or barns where disease occurred previously but the birds were clinically normal at the time of sampling. Based on the observed poultry rearing practices in Kerala, housing system was recorded as extensive (up to 20 birds), semi‐intensive (21 to 200 birds) and intensive (over 200 birds). Backyard birds were usually ‘free range’ in the daytime and housed in wooden cages during the night. In semi‐intensive systems, birds are housed in semi‐permanent to permanent sheds and are not let loose. In intensive systems, birds are usually housed in permanent sheds and are not let loose. Wild birds were characterised as being free range and the one included in this set of samples (Open billed stork (Anastomus oscitans)) was brought to the veterinary clinic for treatment after trauma. The types of poultry sampled included commercial layers and broilers, backyard poultry, ducks, pigeons and turkeys. The samples were collected in brain‐heart infusion broth followed by storage at –80°C until testing.

**TABLE 1:** Apparent prevalence of Newcastle disease virus in Kerala, India

Category	Variable	Positive	Total	Prevalence	95% CI^†^
Vaccination	Yes	129	1573^‡^	8.2	(6.9–9.7)
	No	38	504	7.5	(5.5–10.3)
Flock health status	Healthy	6	650	0.9	(0.4–2.1)
	Mild respiratory signs	32	536	6.0	(4.2–8.4)
	Diseased	129	893	14.5	(12.2–17.0)
Bird health status	Healthy	100	1290	7.8	(6.4–9.4)
	Sick	57	536	10.6	(8.2–13.6)
	Recovered	2	104	1.9	(0.3–7.5)
	Dead	8	149	5.4	(2.5–10.7)
Housing	Backyard	7	125	5.6	(2.5–11.6)
	Free range	0	1	0	(0–97.5)
	Semi‐intensive	13	724	1.8	(1.0–3.1)
	Intensive	147	1229	12.0	(10.2–13.9)
Sex^§^	Female	80	1623	4.9	(4.0–6.1)
	Male	3	93	3.2	(0.8–9.8)
Zone	1	70	1079	6.5	(5.1–8.2)
	2	61	651	9.4	(7.3–11.9)
	3	36	349	10.3	(7.4–14.1)
Bird type	Backyard poultry	5	106	4.7	(1.7–11.2)
	Broilers	84	363	23.1	(19.0–27.9)
	Layers	78	1510	5.2	(4.1–6.4)
	Ducks	0	95	0.00	(0–4.8)
	Pigeons	0	2	0.00	(0–84.2)
	Turkeys	0	2	0.00	(0–84.2)
	Wild birds	0	1	0.00	(0–97.5)

^†^ Estimate and 95% confidence interval calculated by prop.test for denominator >30, by exact binom.test for denominator < 30.

^‡^ The vaccination status of 2 birds could not be ascertained and no NDV was detected.

^§^ The sex of broilers (*n* = 363) was not determined with detection of NDV in 84 samples.

### 2.2 RNA extraction and real‐time reverse transcription‐PCR (RT‐PCR)

Total RNA was extracted from specimens using MagMAX™ 96 AI/ND Viral RNA isolation kit (Thermo Fischer Scientific, Vilnius, Lithuania). Real‐time reverse transcription‐PCR (RT‐PCR) was performed to detect the M gene of NDV using OneStep RT‐PCR kit (Qiagen, Hilden, Germany) and previously published primers (Wise et al., 2004). Based on standard controls, a *C_t_* value of 35 and below was regarded as positive. In order to assess whether the M gene‐positive samples contained lentogenic or mesogenic/velogenic NDV, all these samples were tested by RT‐PCR targeting the F gene of mesogenic/velogenic NDV APMV‐1 using previously published primers (Farkas et al., 2009). Any sample with a *C_t_* value of 40 or below was regarded as positive.

#### 2.2.1 Statistical analysis

Apparent prevalence of NDV and two proportion tests with accompanying 95% confidence intervals (CIs) were calculated with the prop.test function in the stats package in R (https://www.R‐project.org/) as long as the denominator was greater than 30. If it was less than 30 then the binom.test function was used. The R package epitools was used to calculate pairwise risk ratios and 95% CIs using the risk ratio function (https://cran.r‐project.org/web/packages/epitools/index.html). Relative risk (RR) was assessed with respect to various parameters such as vaccination, flock status, bird status, housing, sex, zone, and bird type. The relative risk values between two variables were calculated by the formula [(RR of Var1) – 1] + [1 – (RR of Var2)] = percentage difference between the 2 variables, where Var1 (RR higher than 1) and Var2 (RR lower than 1).

A series of logistic regression models were run using the glm() function R. Of the original 2079 samples, data from 1978 samples were used for the analysis. The 101 samples that were not included in this analysis were from ducks, which were all reared under only one type of housing so these samples contributed to collinearity within the model. The outcome was a positive M gene test and independent variables (risk factors) such as vaccination, migratory season, occurrence of ND outbreaks near the sampling location, bird type, and housing type. Collinearity in the model was ascertained using the vif() function. The log odds ratio output from the glm() was converted into a relative risk score and accompanying 95% confidence interval using the function odds_to_rr() from the sjstats package. This function uses the equation: RR = OR/[(1 – P0) + (P0 × OR)] from Zhang and Yu (1998), Wang (2013) and Grant (2014).

## 3 RESULTS

### 3.1 Clinical signs

**FIGURE 2:**
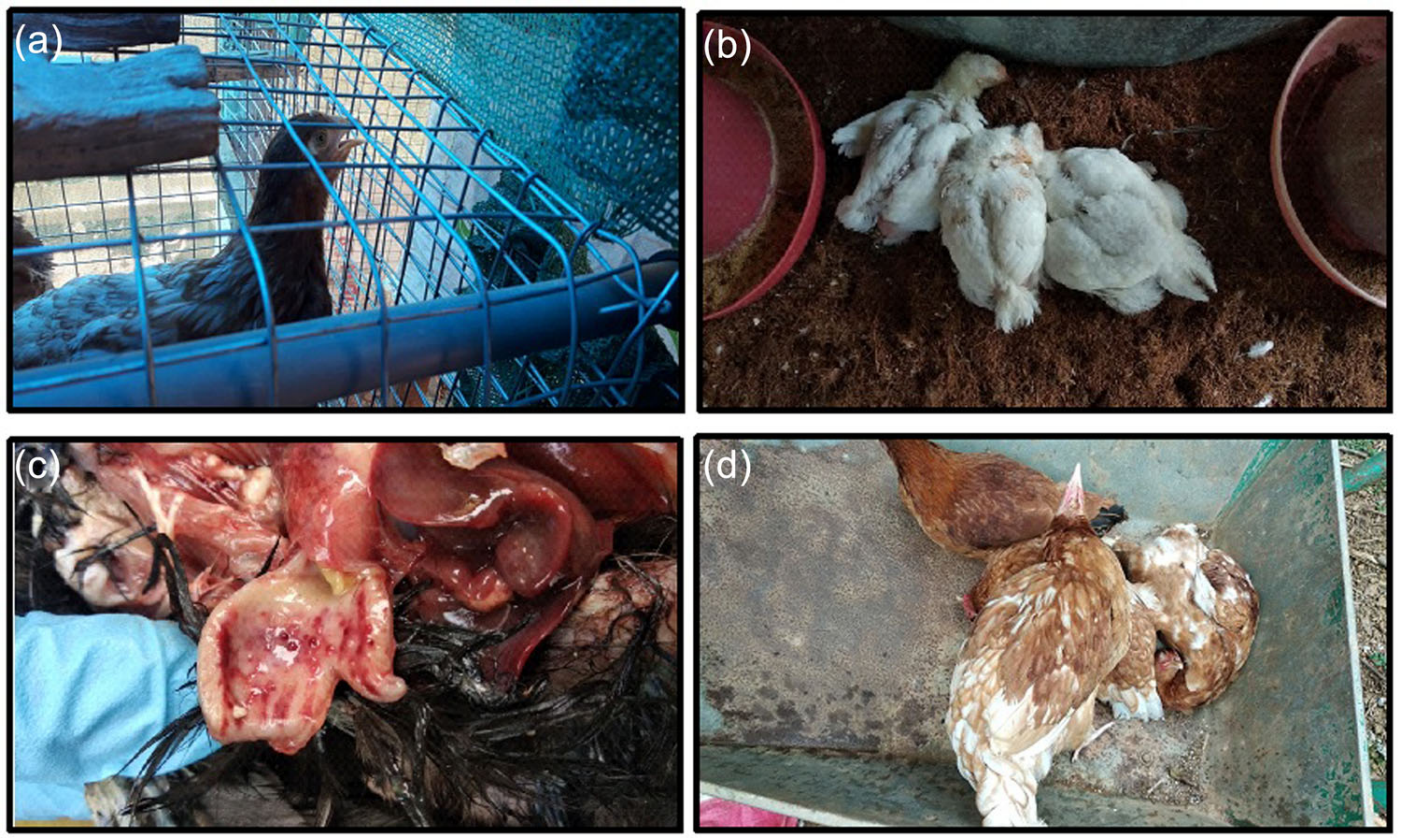
Clinical signs and postmortem lesion observed in affected birds. A layer exhibiting difficulty in respiration (a), huddling of birds (b), haemorrhage at the tip of the proventricular glands and coalesced haemorrhages in proventricular mucosa (c), birds with torticollis (d)

During the course of the study, samples were collected from cases of respiratory illness suggestive of ND. The clinical signs exhibited by the affected birds ranged from mild respiratory distress to difficulty in respiration (Figure 2a) and huddling with other birds (Figure 2b). On postmortem examination, haemorrhage at the tip of the proventricular glands (Figure 2c) and in the cecal tonsils were observed and in some rare cases, torticollis (Figure 2d) and greenish diarrhoea were also observed.

### 3.2 Molecular detection of NDV based on M and F gene‐based RT‐PCR

Of the 70 facilities sampled, NDV was detected in 18 (25.7%, 95% CI: 16.3–37.8). Of the 132 flocks tested, NDV was detected in 21 (15.9%, 95% CI: 10.3–23.5). Of the 2079 samples tested, 167 were positive for the M gene, of which only 7 (4.2%, 95% CI: 1.8–8.8) were positive for the F gene. Of the 167 positive samples, 160 were from choanal swabs, 3 samples were from trachea and 2 each were from lungs and spleen.

Of the 2079 samples tested, 1573 were from vaccinated birds and 504 were from unvaccinated birds (Table 1). The vaccination status of two birds could not be ascertained. Although a majority of the M gene‐positive samples were from vaccinated birds (129 of 1573 vaccinated samples; 8.2%, 95% CI: 6.9–9.7) versus non‐vaccinated birds (38 of 504 unvaccinated samples; 7.5%, 95% CI: 5.5–10.3), the difference between the two proportions was not statistically significant (*p* = 0.7).

Of the 893 samples collected from diseased flocks, 129 were positive for NDV (14.5%, 95% CI: 12.2–17.0). Prevalence was also calculated with respect to the individual bird's health status at the time of sampling. Of the 1290 healthy birds sampled, 100 were M gene positive (7.8%, 95% CI: 6.4–9.4) while 57 of 536 sampled sick birds were positive (10.6%, 95% CI: 8.2–13.6) and there was not a significant difference in the proportion of positive samples in both these categories of birds (*p* = 0.06). The prevalence of the virus in dead birds was 5.4% (95% CI: 2.5–10.7) and in recovered birds it was much lower (1.9%, 95% CI: 0.3–7.5). There was no statistical difference in prevalence between females (4.9%, 95% CI: 4.0–6.1) and males (3.2%, 95% CI: 0.8–9.8).

Prevalence of NDV was highest in intensive housing (12.0%, 95% CI: 10.2–13.9) followed by backyard (5.6%, 95% CI: 2.5–11.6) and semi‐intensive housing (1.8%, 95% CI: 1.0–3.1). Prevalence was highest in zone 3 (10.3%, 95% CI: 7.4–14.1) followed by zones 2 (9.4%, 95% CI: 7.3–11.9) and 1 (6.5%, 95% CI: 5.1–8.2). The highest prevalence was observed in broilers (23.1%, 95% CI: 19.0–27.9) followed by layers (5.2%, 95% CI: 4.1–6.4) and backyard birds (4.7%, 95% CI: 1.7–11.9).

### 3.3 Assessment of associated risk factors for NDV infection

**FIGURE 3:**
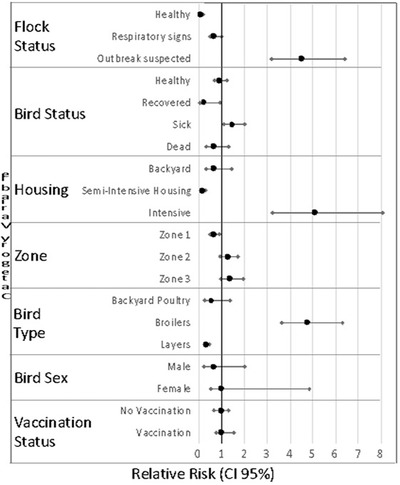
Univariable relative risk of a positive M‐gene test for Newcastle disease virus by risk factor variables

In India, NDV control is primarily with vaccination and varies with bird age and rearing conditions. Hence, the relative risk (RR) was assessed for specific risk factors including: vaccination status, flock health status, bird health status, housing, sex, zone and bird type (Table 2; Figure 3). In pairwise comparisons, no significant difference was observed between the infection risk associated with vaccinated and unvaccinated birds (RR: 1.1, 95% CI: 0.8–1.5) and 0.9 (95% CI: 0.7–1.3). Notably, there was a higher risk for diseased flocks when compared to healthy flocks (RR: 15.6, 95% CI: 7.0–35.2). Further, the RR of flocks exhibiting mild respiratory signs was 6.5 (95% CI: 2.7–15.4) when compared to healthy flocks, which confirms respiratory symptoms to be a significant risk factor for NDV infection. Birds in diseased flocks were 2.4 times more likely to be infected with NDV as compared to those with mild respiratory signs (RR: 2.4, 95% CI: 1.7–3.5).

**TABLE 2:** Relative risk for each variable compared to the other individual variables within that category and 95% confidence interval (CI)

Category	Comparison of variables	Relative risk	95% CI^†^
Vaccination	Yes	1.1	(0.8–1.5)
	No	0.9	(0.7–1.3)
Flock status	Healthy vs. diseased *	0.06	(0.03–0.1)
	Diseased vs. healthy*	15.6	(7.0–35.2)
	Healthy vs. mild respiratory signs*	0.2	(0.07–0.4)
	Mild respiratory signs vs. healthy*	6.5	(2.7–15.4)
	Diseased vs. mild respiratory signs *	2.4	(1.7–3.5)
	Mild respiratory signs vs. diseased*	0.4	(0.3–0.6)
Bird status	Healthy vs. dead	1.4	(0.7–2.9)
	Dead vs. healthy	0.7	(0.3–1.4)
	Healthy vs. recovered*	4.0	(1.0–16.1)
	Recovered vs. healthy	0.2	(0.06–1.0)
	Healthy vs. sick	0.7	(0.5–1.0)
	Sick vs. healthy*	1.4	(1.0–1.9)
	Dead vs. recovered	2.8	(0.6–12.9)
	Recovered vs. dead	0.4	(0.08–1.7)
	Dead vs. sick	0.5	(0.3–1.0)
	Sick vs. dead	2.0	(1.0–4.1)
	Recovered vs. sick*	0.2	(0.04–0.7)
	Sick vs. recovered*	5.5	(1.4–22.3)
Housing	Backyard vs. intensive*	0.5	(0.2–1.0)
	Intensive vs. backyard*	2.1	(1.0–4.5)
	Backyard vs. semi*	3.1	(1.3–7.7)
	Semi vs. backyard*	0.3	(0.1–0.8)
	Intensive vs. semi*	6.7	(3.8–11.7)
	Semi vs. intensive*	0.2	(0.09–0.3)
Sex	Female	2.8	(0.9–8.7)
	Male	0.4	(0.1–1.1)
Zone	1 vs. 2*	0.7	(0.5–1.0)
	2 vs. 1*	1.4	(1.0–2.0)
	1 vs. 3*	0.6	(0.4–0.9)
	3 vs. 1*	1.6	(1.1–2.3)
	2 vs. 3	0.9	(0.6–1.3)
	3 vs. 2	1.1	(0.8–1.6)
Bird type	Backyard vs. broiler*	0.2	(0.09–0.5)
	Broiler vs. backyard*	4.9	(2.1–11.8)
	Backyard vs. layer	0.9	(0.48–2.2)
	Layer vs. backyard	1.1	(0.5–2.6)
	Broiler vs. layer*	4.5	(3.4–5.9)
	Layer vs. broiler*	0.2	(0.2–0.3)

^†^ Calculated with normal approximation (Wald) with small sample adjustment.

* Statistically significant (confidence intervals do not cross 1).

At an individual bird level, healthy birds were four times as likely to be M gene‐positive than recovered birds (RR: 4.0, 95% CI: 1.0–16.1). The risk of a positive test among sick birds was also twice the risk among dead birds (RR: 2.0, 95% CI: 1.0–4.1). Additionally, sick birds had a risk five times that of recovered birds (RR: 5.5, 95% CI: 1.4–22.3). Birds in intensive housing were twice as likely to be M gene‐positive compared to those reared under backyard conditions (RR: 2.1, 95% CI: 1.0–4.5) and nearly seven times as likely when compared to those in semi‐intensive conditions (RR: 6.7, 95% CI: 3.8–11.7). There was no statistically significant risk associated with the sex of the birds although a vast majority of samples was taken from female birds; the RR of a positive test among female birds was 2.8 (95% CI: 0.9–8.7). When the infection rate in each of the three zones was compared with the other two, the relative risk of a bird in zone 1 getting infected was lower (RR: 0.7, 95% CI: 0.5–1.0) than that in zone 2 (RR: 1.4, 95% CI: 1.0–2.0) and zone 3 (RR: 1.6, 95% CI: 1.1–2.3). The risk to broilers was nearly five times higher that of backyard birds or layers in pairwise comparisons (RR: 4.9, 95% CI: 2.1–11.8 and RR: 4.5 95% CI: 3.4–5.9, respectively).

**FIGURE 4:**
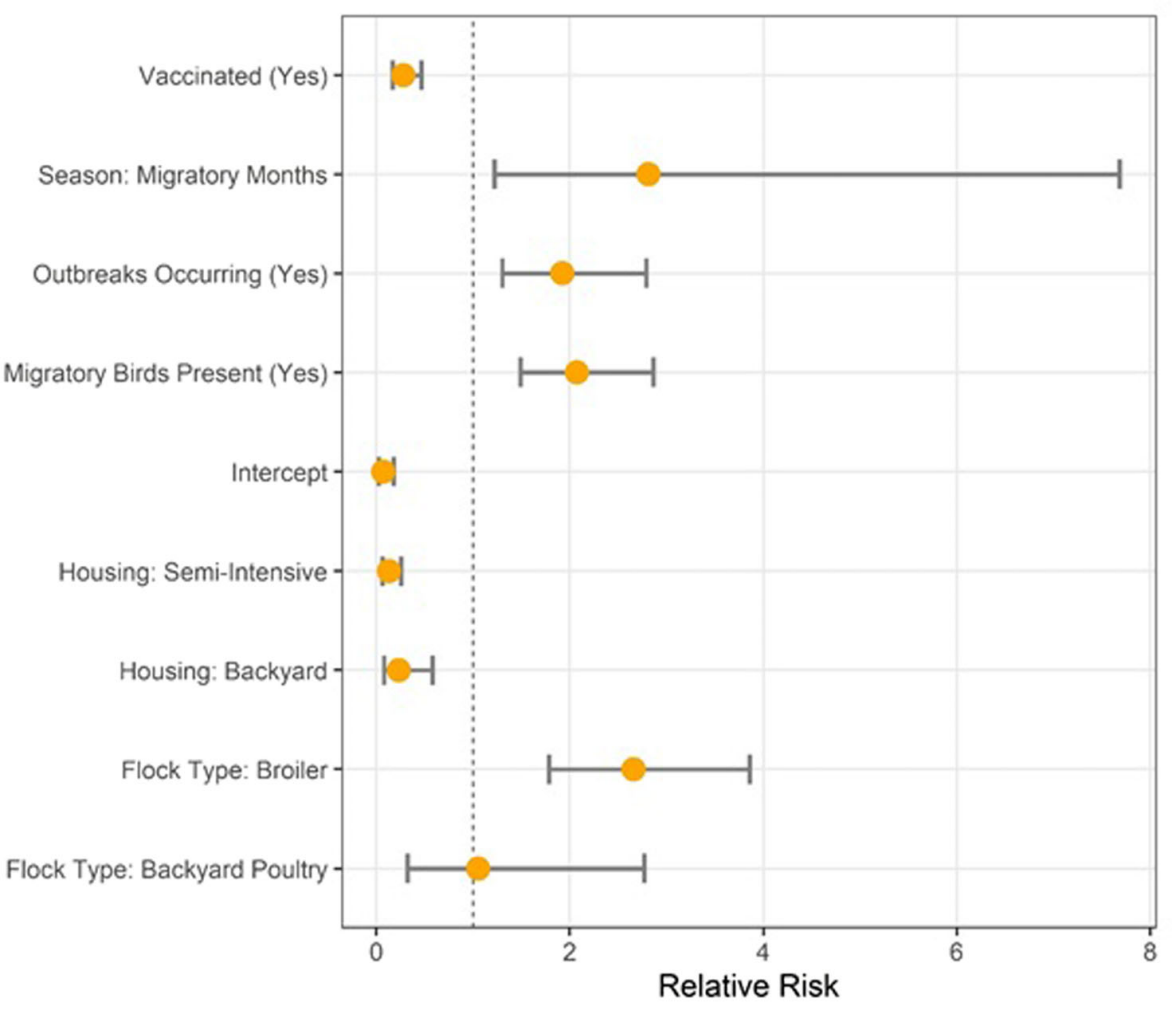
Relative risk of Newcastle disease virus infection (M‐gene positive) by risk factor from multivariable logistic regression analysis

The results of the multivariable analysis are shown in Table 3 and Figure 4. The analysis shows that, in contrast to the result of univariate analysis, there was a significantly lower risk of M gene positive result among vaccinated birds, indicating a protective effect with odds ratio (OR) and risk ratio (RR) of 0.3, respectively (RR 95% CI: 0.2–0.5). Significantly higher risk was observed in samples collected during migratory season (OR: 2.9; RR: 2.8; RR 95% CI: 1.2–7.7), if migratory birds were seen near the facility (OR: 2.2; RR: 2.1; RR 95% CI: 1.2–7.7) and if there was a history of NDV outbreaks occurring nearby (OR 2.1; RR: 1.9; RR 95% CI: 1.3–2.8). As was observed in pairwise comparisons, multivariable analysis showed that backyard (OR: 0.2; RR: 0.2; RR 95% CI: 0.08–0.6) and semi‐intensive housing (OR: 0.1; RR: 0.1; RR 95% CI: 0.06–0.3) had a significant protective effect when compared to intensive housing. Lastly, broilers were at a higher risk (OR 2.9; RR 2.7; RR 95% CI 1.8–3.9) of being infected as compared to layers, but backyard birds were not at significantly higher risk than layers (OR: 1.1; RR: 1.1; RR 95% CI: 0.3–2.8).

**TABLE 3:** Odds ratio, relative risk and relative risk confidence interval (CI) for risk factors of ND prevalence from multivariable logistic regression

Parameter	Odds ratio	Relative risk	Relative risk 95% CI
Intercept	0.1	0.1	(0.02–0.2)
Vaccination	0.3	0.3	(0.2–0.5)
Housing: backyard	0.2	0.2	(0.08–0.6)
Housing: Semi‐intensive	0.1	0.1	(0.06–0.3)
NDV outbreaks	2.1	1.9	(1.3–2.8)
Presence of migratory birds	2.2	2.1	(1.5–2.9)
Migratory season	2.9	2.8	(1.2–7.7)
Bird type: backyard poultry	1.1	1.1	(0.3–2.8)
Bird type: broiler	2.9	2.7	(1.8–3.9)

## 4 DISCUSSION

The present study was carried out to assess the prevalence of NDV among different types of poultry reared under different management and housing conditions across Kerala. Other parameters considered were vaccination, condition of the flock, bird health, vaccination, sex and zone. Time of sampling such as migratory and non‐migratory season, presence of migratory birds in the vicinity and presence and history of ND outbreaks in the surrounding areas were also considered. Analysis was performed at the flock‐level as well as at the individual bird level. Both univariate and multivariate analyses were performed.

NDV was detected in flocks of broilers, layers and backyard poultry by M gene‐based RT‐PCR. The virus was detected to a greater extent in flocks exhibiting signs of respiratory disease. Additionally, NDV was detected in individual birds that were apparently healthy, sick and those reared under intensive housing. The prevalence was higher in broilers, which were under the intensive system of housing. Though the NDV prevalence in vaccinated flocks was slightly higher than in unvaccinated flocks, this was not statistically significant. Multivariable analysis revealed a significantly increased risk of NDV detection among samples collected during the migratory season, when migratory birds were nearby the facility sampled, and when NDV outbreaks were occurring nearby. In contrast to the univariable analysis, multivariable analysis revealed that vaccination had a protective effect on the birds. In both analyses, higher risk of NDV detection was associated with the intensive system of housing.

In Kerala, the primary vaccination strategy for ND in commercial layers is the use of a lentogenic strain of NDV at the first week of age, followed by a booster with a mesogenic strain (e.g., R2B) at two months of age. However, it is worth mentioning that the currently used vaccine strains were isolated three to seven decades earlier and are regarded as genetically distinct from the currently circulating virulent NDV strains. The high genetic distance between the vaccine and the current NDV strains prevents effective reduction of shedding of virulent virus from vaccinated birds (Dimitrov et al., 2017). However, results of the multivariable analysis indicate that vaccination did have a protective effect.

The M gene primers pick up vaccine strains that are largely (but not exclusively) lentogenic in nature. Hence, F‐gene primers and probes were used to determine the prevalence of mesogenic/velogenic (M/V) strains. The low prevalence of M/V pathotypes suggests that a vast majority of circulating strains are probably lentogenic in nature. Since lentogenic strains were equally prevalent in non‐vaccinated flocks, it can be presumed that at least some of them were wild‐type strains. Another reason for reduced detection of mesogenic/velogenic NDV may be due to the lack of sensitivity of the primers and probes for virulent NDV prevalent in India. However, to preclude any issues with sensitivity and specificity of the PCR‐based pathotyping approach, future molecular epidemiologic and phylogenomic analyses are planned.

The occurrence of NDV in vaccinated birds has been previously reported in several other countries in Asia, Africa and Central America where NDV is endemic (Dey et al., 2014). Although vaccination protects against clinical disease, it fails to protect against viral shedding when birds are challenged with a genotype different than that contained in the vaccine (Kapczynski & King, 2005; Miller et al., 2007). For example, a new subtype of virulent genotype XIII has been shown to cause severe outbreaks in vaccinated commercial broiler farms in Tamil Nadu, Southern India (Gowthaman, Ganeshan et al., 2019). Additionally, mixed infection with both vaccine and field strains in a flock is also possible. The failure of vaccination to protect a flock can be due not only to antigenic variation or genotype mismatch with circulating strains but also to poor flock immunity because of inadequate vaccination that can be attributed to poor vaccine storage conditions or faulty vaccine administration (Dortmans et al., 2012; Liu et al., 2018).

In the present study, NDV was found to be more prevalent in broilers (23.1%, 95% CI: 19.0–27.9) than in layers (5.2%, 95% CI: 4.1–6.4) or backyard birds (4.7%, 95% CI: 1.7–11.2). The broilers also had a higher RR (univariable analysis, Figure 3). Higher prevalence of NDV in broilers as compared to layers has been reported previously (Rahman et al., 2017). The greater chance of birds reared under intensive systems contracting viral infections has been reported previously. However, this system allowed for better control of disease. On the other hand, the control of diseases in small, rural, extensive poultry flocks in developing countries was difficult and that the incidence of diseases, such as ND, in these birds may represent a threat to intensively managed systems (Biggs, 1982).

Most of the clinical signs observed during sample collection were typical of ND (e.g., respiratory distress, ruffled feathers, and high morbidity). During necropsy, haemorrhages in the proventriculus and caecal tonsils were often observed, which are suggestive of ND. Similar observations have been reported in NDV outbreaks in Gujarat, India (Khorajiya et al., 2015). In the present study, samples collected from birds with clinical signs of respiratory illness and high morbidity were positive for NDV. However, there are other diseases, which can present a clinical picture similar to that of ND, for example, infectious bronchitis, infectious laryngotracheitis and avian influenza. In a recent study, co‐infection of flocks with low‐pathogenic avian influenza virus (AIV) and virulent NDV was reported and it was concluded that AIV may help increase the severity of NDV in layers (Gowthaman, Singh et al., 2019). It is important, therefore, that these disease conditions are differentially diagnosed. As a part of differential diagnosis, we tested a few samples for avian infectious bronchitis virus (IBV) and NDV and found some of them to be positive for IBV and negative for NDV (data not shown). Hence, respiratory signs exhibited by the birds can also be due to IBV. But viral infectious diseases such as infectious laryngotracheitis and pneumovirus infection are not currently known to pose a significant threat to poultry industry in Kerala. However, to rule out the possible contributory role of these respiratory viruses, selected samples from positive flock are being analysed by Next Generation Sequencing.

This study has several limitations. First, although this analysis suggests a higher number of outbreaks in zone 1, this may not give an accurate picture of the NDV prevalence in zones 2 and 3 as they were far away from the sampling centre location and information on outbreak occurrence in these areas may be under‐reported. This study considers that the primers for M and F gene are capable of detecting prevalent NDV strains. This might not be the case as variant strains may be present in this region. This seems to be especially true in the case of F gene testing which gave a very low positivity rate. Lastly, non‐random sampling is a limitation of the study for generalisation of results but does not affect the relationships observed between infection and risk factors.

On the contrary, during the study we have also observed that cases that appeared to be ND were not detected by the M or F gene primers. It has been reported that techniques based on probe/primer hybridisation to a specific site are very sensitive to mismatches that often produce false‐negative results (Cattoli et al., 2009; Kim et al., 2006). The M and F gene primers used in this study were described in 2004 and 2009, respectively, and there has been significant additions in the genomic data of NDV in the databases and new genotypes have been described which might not be detected by the primer/probes used. In addition, the authors who have designed the M gene primer in the assay have reported that the oligos were not an exact match to all APMV‐1 isolates whose sequence is available in the databases at that time (Wise et al., 2004). Failures in detection of NDV based on M and F gene based diagnostic primers have led to description of diagnostic assays based on other regions of the virus (Ferreira & Suarez, 2019). Lastly, vaccination history is self‐reported by the owner and there are no records to prove that a flock is vaccinated or not and this may bias the observed results.

In conclusion, this study detected NDV in different types of poultry reared in Kerala, which suggests that the virus is endemic in the State. Birds reared under intensive conditions were at more risk of NDV infection. Vaccination did have a protective effect on the occurrence of NDV infection. Studies on F gene‐based characterisation and next generation sequencing are underway to characterise different genotypes and sub‐genotypes of NDV present in poultry in Kerala to help develop better diagnostic strategies to detect and differentiate virulent NDV, if any, in Kerala. Overall, the results of this study provide a framework for future longitudinal studies on NDV in various types of birds in Kerala and other geographies where the virus may be endemic.

## ETHICS STATEMENT

The study was undertaken after obtaining permission from Institutional Animal Ethics Committee (IAEC) of College of Veterinary and Animal Sciences, Pookode, Kerala (Approval Number: IAEC/COVAS/PKD/2/2017).

## AUTHOR CONTRIBUTIONS

Chintu Ravishankar: conceptualisation; investigation; methodology; project administration; supervision; validation; writing – original draft; writing – review & editing. Ravindran Rajasekhar: investigation; writing – review & editing. Anneth Alice John: investigation; writing – review & editing. Nithin Divakar: investigation; writing – review & editing. George Chandy: investigation; writing – review & editing. Deepika Chaudhary: investigation; writing – review & editing. Renu Singh: investigation; writing – review & editing. Niranjana Sahoo: project administration; writing – review & editing. Sunil K. Mor: conceptualisation; funding acquisition; methodology; writing – review & editing. Nand K. Mahajan: project administration; supervision; writing – review & editing. Naresh Jindal: project administration; supervision; writing – review & editing. Megan A. Schilling: formal analysis; writing – review & editing. Catherine M. Herzog: data curation; formal analysis; software; validation; visualisation; writing – review & editing. Saurabh Basu: software. Jessica Radzio‐Basu: data curation; formal analysis; software; visualisation; writing – review & editing.

### PEER REVIEW

The peer review history for this article is available at https://publons.com/publon/10.1002/vms3.747

## Supporting information



Supporting Information

Supporting Information

## Data Availability

The data that supports the findings of this study are available in the supplementary material of this article
