# Supplementary material for: Detection of Newcastle disease virus and assessment of associated relative risk in backyard and commercial poultry in Kerala, India
Source: Vet Med Sci. 2022 Feb 24;8(3):1146–56. doi: 10.1002/vms3.747 (PMC9122440; doi:10.1002/vms3.747)
Supplement: Supplementary file 1 — Supporting Information [file VMS3-8-1146-s001.docx]

**Supplementary Table 1. Details of flock size, samples collected per flock and flock sampling density**

| **Flock ID** | **No. of birds sampled (n)** | **Flock size (N)** | **Flock sample density (n/N)** |
| --- | --- | --- | --- |
| Flock 1 | 30 | 125 | 0.240 |
| Flock 2 | 104 | 8000 | 0.013 |
| Flock 3 | 43 | 600 | 0.072 |
| Flock 4 | 2 | 4 | 0.500 |
| Flock 5 | 1 | 150 | 0.007 |
| Flock 6 | 3 | 9 | 0.333 |
| Flock 7 | 7 | 30 | 0.233 |
| Flock 8 | 12 | 2000 | 0.006 |
| Flock 9 | 6 | 20 | 0.300 |
| Flock 10 | 1 | 10 | 0.100 |
| Flock 11 | 3 | 60 | 0.050 |
| Flock 12 | 5 | 15 | 0.333 |
| Flock 13 | 3 | 500 | 0.006 |
| Flock 14 | 2 | 2 | 1.000 |
| Flock 15 | 12 | 18 | 0.667 |
| Flock 16 | 33 | 800 | 0.041 |
| Flock 17 | 12 | 1300 | 0.009 |
| Flock 18 | 2 | 9 | 0.222 |
| Flock 19 | 5 | 155 | 0.032 |
| Flock 20 | 11 | 60 | 0.183 |
| Flock 21 | 3 | 20 | 0.150 |
| Flock 22 | 15 | 30 | 0.500 |
| Flock 23 | 14 | 40 | 0.350 |
| Flock 24 | 1 | 2 | 0.500 |
| Flock 25 | 3 | 30 | 0.100 |
| Flock 26 | 17 | 155 | 0.110 |
| Flock 27 | 9 | 60 | 0.150 |
| Flock 28 | 71 | 600 | 0.118 |
| Flock 29 | 13 | 30 | 0.433 |
| Flock 30 | 7 | 30 | 0.233 |
| Flock 31 | 4 | 50 | 0.080 |
| Flock 32 | 1 | 155 | 0.006 |
| Flock 33 | 3 | 17 | 0.176 |
| Flock 34 | 12 | 35 | 0.343 |
| Flock 35 | 6 | 9 | 0.667 |
| Flock 36 | 1 | 300 | 0.003 |
| Flock 37 | 20 | 150 | 0.133 |
| Flock 38 | 20 | 800 | 0.025 |
| Flock 39 | 5 | 280 | 0.018 |
| Flock 40 | 10 | 98 | 0.102 |
| Flock 41 | 19 | 200 | 0.095 |
| Flock 42 | 33 | 94 | 0.351 |
| Flock 43 | 59 | 360 | 0.164 |
| Flock 44 | 63 | 250 | 0.252 |
| Flock 45 | 1 | 9 | 0.111 |
| Flock 46 | 4 | 16 | 0.250 |
| Flock 47 | 4 | 50 | 0.080 |
| Flock 48 | 3 | 40 | 0.075 |
| Flock 49 | 5 | 60 | 0.083 |
| Flock 50 | 20 | 200 | 0.100 |
| Flock 51 | 9 | 5000 | 0.002 |
| Flock 52 | 50 | 1200 | 0.042 |
| Flock 53 | 12 | 30 | 0.400 |
| Flock 54 | 121 | 1000 | 0.121 |
| Flock 55 | 2 | 2 | 1.000 |
| Flock 56 | 10 | 64 | 0.156 |
| Flock 57 | 2 | 155 | 0.013 |
| Flock 58 | 20 | 105 | 0.190 |
| Flock 59 | 9 | 25 | 0.360 |
| Flock 60 | 4 | 30 | 0.133 |
| Flock 61 | 5 | 20 | 0.250 |
| Flock 62 | 10 | 20 | 0.500 |
| Flock 63 | 2 | 30 | 0.067 |
| Flock 64 | 8 | 300 | 0.027 |
| Flock 65 | 11 | 16 | 0.688 |
| Flock 66 | 3 | 9 | 0.333 |
| Flock 67 | 10 | 148 | 0.068 |
| Flock 68 | 108 | 6000 | 0.018 |
| Flock 69 | 30 | 800 | 0.038 |
| Flock 70 | 54 | 65 | 0.831 |
| Flock 71 | 14 | 125 | 0.112 |
| Flock 72 | 13 | 200 | 0.065 |
| Flock 73 | 10 | 190 | 0.053 |
| Flock 74 | 4 | 60 | 0.067 |
| Flock 75 | 9 | 60 | 0.150 |
| Flock 76 | 17 | 60 | 0.283 |
| Flock 77 | 65 | 5000 | 0.013 |
| Flock 78 | 1 | 150 | 0.007 |
| Flock 79 | 5 | 30 | 0.167 |
| Flock 80 | 10 | 250 | 0.040 |
| Flock 81 | 3 | 155 | 0.019 |
| Flock 82 | 1 | 1 | 1.000 |
| Flock 83 | 5 | 78 | 0.064 |
| Flock 84 | 5 | 200 | 0.025 |
| Flock 85 | 2 | 17 | 0.118 |
| Flock 86 | 8 | 30 | 0.267 |
| Flock 87 | 2 | 10 | 0.200 |
| Flock 88 | 1 | 2 | 0.500 |
| Flock 89 | 5 | 30 | 0.167 |
| Flock 90 | 12 | 50 | 0.240 |
| Flock 91 | 20 | 60 | 0.333 |
| Flock 92 | 12 | 2000 | 0.006 |
| Flock 93 | 6 | 30 | 0.200 |
| Flock 94 | 11 | 500 | 0.022 |
| Flock 95 | 8 | 10 | 0.800 |
| Flock 96 | 3 | 20 | 0.150 |
| Flock 97 | 10 | 3900 | 0.003 |
| Flock 98 | 39 | 75 | 0.520 |
| Flock 99 | 50 | 2250 | 0.022 |
| Flock 100 | 12 | 15 | 0.800 |
| Flock 101 | 101 | 1500 | 0.067 |
| Flock 102 | 20 | 300 | 0.067 |
| Flock 103 | 4 | 54 | 0.074 |
| Flock 104 | 24 | 60 | 0.400 |
| Flock 105 | 22 | 480 | 0.046 |
| Flock 106 | 64 | 2500 | 0.026 |
| Flock 107 | 3 | 30 | 0.100 |
| Flock 108 | 1 | 200 | 0.005 |
| Flock 109 | 10 | 80 | 0.125 |
| Flock 110 | 5 | 5 | 1.000 |
| Flock 111 | 8 | 60 | 0.133 |
| Flock 112 | 1 | 10 | 0.100 |
| Flock 113 | 1 | 1 | 1.000 |
| Flock 114 | 2 | 300 | 0.007 |
| Flock 115 | 2 | 20 | 0.100 |
| Flock 116 | 10 | 184 | 0.054 |
| Flock 117 | 4 | 17 | 0.235 |
| Flock 118 | 10 | 154 | 0.065 |
| Flock 119 | 1 | 50 | 0.020 |
| Flock 120 | 10 | 30 | 0.333 |
| Flock 121 | 31 | 1863 | 0.017 |
| Flock 122 | 2 | 2 | 1.000 |
| Flock 123 | 4 | 60 | 0.067 |
| Flock 124 | 5 | 500 | 0.010 |
| Flock 125 | 5 | 20 | 0.250 |
| Flock 126 | 49 | 170 | 0.288 |
| Flock 127 | 50 | 1720 | 0.029 |
| Flock 128 | 6 | 1000 | 0.006 |
| Flock 129 | 14 | 20 | 0.700 |
| Flock 130 | 11 | 154 | 0.071 |
| Flock 131 | 5 | 2000 | 0.003 |
| Flock 132 | 8 | 20 | 0.400 |
